# Trends in substitution models of molecular evolution

**DOI:** 10.3389/fgene.2015.00319

**Published:** 2015-10-26

**Authors:** Miguel Arenas

**Affiliations:** Institute of Molecular Pathology and Immunology of the University of PortoPorto, Portugal

**Keywords:** substitution model, molecular evolution, model selection, molecular adaptation, phylogenetics, phylogenomics

## Abstract

Substitution models of evolution describe the process of genetic variation through fixed mutations and constitute the basis of the evolutionary analysis at the molecular level. Almost 40 years after the development of first substitution models, highly sophisticated, and data-specific substitution models continue emerging with the aim of better mimicking real evolutionary processes. Here I describe current trends in substitution models of DNA, codon and amino acid sequence evolution, including advantages and pitfalls of the most popular models. The perspective concludes that despite the large number of currently available substitution models, further research is required for more realistic modeling, especially for DNA coding and amino acid data. Additionally, the development of more accurate complex models should be coupled with new implementations and improvements of methods and frameworks for substitution model selection and downstream evolutionary analysis.

## Introduction

Substitution models of evolution describe the rates of change of fixed mutations among sequences and constitute the basis of the evolutionary analysis of genetic data at the molecular level. Although, the first substitution models that corrected the effects of multiple replacements were documented a long time ago (Jukes and Cantor, 1969; Dayhoff et al., 1978; Kimura, 1980), they were not applied in evolutionary analysis until much later when they were implemented in phylogenetic software packages based on maximum-likelihood (ML) methods (Felsenstein, 1991; Swofford, 1993). Then, it was noted that substitution model misspecification might bias phylogenetic inferences (Posada and Crandall, 2001; Minin et al., 2003; Lemmon and Moriarty, 2004). As a consequence, the selection of the best-fit substitution model became an essential stage in the pipeline of phylogenetic inference (Posada and Crandall, 1998, 2001), which also increased the popularity of substitution models in phylogenetics.

Nowadays, substitution models are routinely used in diverse areas of evolutionary biology. A large number of substitution models exist and new models are emerging to mimic the evolution of particularly complex real datasets. Nevertheless, recent studies suggest that there is still room for improving the commonly used substitution models (e.g., Keane et al., 2006).

This perspective provides an overview on the trends of substitution models of DNA, codon and amino acid evolution, including goals, and pitfalls of well-established substitution models. It also discusses directions for future research in substitution models of evolution and the establishment of complex substitution models as an essential step for phylogenetic inference.

## Trends in DNA substitution models

The first and simplest model to mimic the DNA substitution process was described by Jukes and Cantor (JC) (1969). This model considers one rate of change between all nucleotides and equal nucleotide frequencies. However, changes between bases with equal chemical nature (transitions) are more common than changes between bases with different chemical structure (transversions) because the replacement of a similar structure is more likely in terms of molecular energy. Moreover, the genetic code allows for more transitions than transversions without amino acid replacement (Kimura, 1980; Collins and Jukes, 1994). Motivated by this evidence, Kimura (1980) presented the two-parameter model (K80), where the rates of change differ between transitions and transversions. Similarly, Felsenstein (1981) (F81) extended the JC model to include different nucleotide frequencies, which can also appear as a consequence of the physicochemical properties of nucleotides and the operation of natural selection. A number of models were later developed by incorporating extensions to those original models [i.e., HKY (Hasegawa et al., 1985) and SYM (Zharkikh, 1994)]. Following this trend, the most complex model, the general time-reversible model (GTR; Tavaré, 1986), incorporates different rates for every change and different nucleotide frequencies. In addition, a proportion of invariable sites (+I) (Shoemaker and Fitch, 1989) and/or rate of variation across sites (+G) (Yang, 1994) can be incorporated into any model. For technical details about these parametric DNA substitution models the reader is referred to the following reviews (Liò and Goldman, 1998; Felsenstein, 2004).

The stationary, reversible and homogeneous DNA substitution models derived from all possible combinations (equal/different rates of change and frequencies, with/without +G and/or +I; more than 1600 models) have already been defined, and are currently implemented in some phylogenetic programs (e.g., Darriba et al., 2012; Arenas and Posada, 2014b), see also Table 1. However, despite the large number of available DNA substitution models, the GTR +G +I model usually fits real data better than the other (simpler) alternative models (Sumner et al., 2012b), suggesting that the evolutionary process is very complex. Importantly, the GTR model presents undesirable mathematical properties [i.e., the multiplication of two GTR matrices does not return another GTR matrix (Sumner et al., 2012a)] for its application as a Markov model in phylogenetics (see Gatto et al., 2006; Sumner et al., 2012a,b). Indeed, the frequent selection of the most complex substitution model may suggest that more complex models could improve the fitting to real data (e.g., Jayaswal et al., 2011).

**Table 1:** **Substitution models implemented in relevant software for phylogenetic inference**.

**Program**	**Task**	**Substitution model**			**Genome**	**Rate variation**	**References**
		**Non-coding**	**Coding**	**Amino acid**			
PhyML	Inference	All	–	Blosum62, CpRev, Dayhoff, FLU, HIVb, HIVw, JTT, LG, Mtart, Mtmam, Mtrev, RtRev, VT, WAG +F	–	+I +G	Guindon et al., 2010
CodonPhyML	Inference	–	GY94^b^, MG94, YAP, ECMs	–	–	+G	Gil et al., 2013
RAxML	Inference	JC, K80, HKY, GTR	Nt^a^	Blosum62, CpRev, Dayhoff, DUMMY, FLU, HIVb, HIVw, JTT, JonesDCMUT, LG, Mtart, Mtmam, Mtrev, Mtzoa, PMB, RtRev, STMREV, VT, WAG +F	Partitioned models can be specified	+I +G	Stamatakis, 2006
MEGA	Inference	JC, K2P, HKY, TN93, GTR	NG86	CpRev, Dayhoff, JTT, LG, MtRev, RtRev, WAG	–	+I +G	Tamura et al., 2013
Hyphy	Inference	All	GY94^b^, MG94, ECM	Dayhoff, HIVb, HIVw, JTT, Mtmam, Mtrev, RtRev, WAG +F	Partitioned models can be specified	+I +G	Pond et al., 2005
PAML	Inference	All	GY94^b^^,^ ^c^, ECMs	CpRev, Dayhoff, DayhoffDCMUT, Grantham, JTT, JonesDCMUT, LG, Miyata, Mtart, Mtmam, Mtrev24, Mtzoa, WAG +F	–	+I +G	Yang, 2007
MrBayes and BEST	Inference	All	GY94^b^, MG94	Blosum62, CpRev, Dayhoff, Mtmam, Mtrev, RtRev, VT, WAG +F	Partitioned models can be specified	+I +G	Ronquist et al., 2012
BEAST	Inference	JC, HKY, TN93, GTR	Nt^a^	Blosum62, CpRev, Dayhoff, FLU, JTT, LG, Mtrev, WAG	–	+I +G	Bouckaert et al., 2014
OmegaMap	Inference	–	NY98^b^	–	–	–	Wilson and McVean, 2006
Lamarc	Inference	JC, K2P, F84, GTR	–	–	–	+G	Kuhner, 2006
CodABC	Inference	–	GY94	–	–	+I +G	Arenas et al., 2015a
MySSP	Simulation	All	–	–	–	+G	Rosenberg, 2005
Seq-Gen	Simulation	All	Nt^a^	Blosum62, CpRev, JTT, mtREV, PAM, and WAG +F	–	+I +G	Rambaut and Grassly, 1997
indel-Seq-Gen	Simulation	All	Nt^a^	Blosum62, CpRev, JTT, mtREV, PAM, WAG +F	–	+I +G	Strope et al., 2009
INDELible	Simulation	All	GY94^b^^,^ ^c^, ECMs	Blosum62, CpRev, Dayhoff, DayhoffDCMUT, HIVb, HIVw, JTT, JonesDCMUT, LG, Mtart, Mtmam, Mtrev24, RtRev, VT, WAG +F	–	+I +G	Fletcher and Yang, 2009
EVOLVER	Simulation	All	GY94^b^^,^ ^c^, ECMs	CpRev, Dayhoff, DayhoffDCMUT, Grantham, JTT, JonesDCMUT, LG, Miyata, Mtart, Mtmam, Mtrev24, Mtzoa, WAG +F	–	+I +G	Yang, 2007
Recodon and NetRecodon	Simulation	All	GY94^b^	–	–	+I +G	Arenas and Posada, 2007, 2010
ProteinEvolver	Simulation	All	–	3D structural constraints, Blosum62, CpRev, Dayhoff, DayhoffDCMUT, HIVb, HIVw, JTT, JonesDCMUT, LG, Mtart, Mtmam, Mtrev24, RtRev, VT, WAG +F	–	+I +G	Arenas et al., 2013
CoalEvol	Simulation	All	GY94^b^^,^ ^c^, MG94, ECMs, HB	Blosum62, CpRev, Dayhoff, DayhoffDCMUT, HIVb, HIVw, JTT, JonesDCMUT, LG, Mtart, Mtmam, Mtrev24, RtRev, VT, WAG +F	–	+I +G	Arenas and Posada, 2014b
SIMPROT	Simulation	–	–	PAM, JTT, PMB	–	+G	Pang et al., 2005
PhyloSim	Simulation	All	GY94^b^^,^ ^c^, ECMs	CpRev, JTT, JonesDCMUT, LG, Mtart, Mtmam, Mtrev24, Mtzoa, WAG +F	–	+I +G	Sipos et al., 2011
π BUSS	Simulation	HKY, TN93, GTR	GY94, MG94	BLOSUM, Dayhoff, LG, JTT, WAG +F	–	+I +G	Bielejec et al., 2014
SGWE	Simulation	All	GY94^b^^,^ ^c^, MG94, ECMs, HB	Blosum62, CpRev, Dayhoff, DayhoffDCMUT, HIVb, HIVw, JTT, JonesDCMUT, LG, Mtart, Mtmam, Mtrev24, RtRev, VT, WAG +F	User-specified regions	+I +G	Arenas and Posada, 2014b
ALF	Simulation	F84, HKY, TN93, GTR	GY94^b^, ECMs	Gonnet, JTT, LG, PAM, WAG +F	User-specified regions	+I +G	Dalquen et al., 2012

“*Task” indicates if the program is oriented to perform evolutionary inference or simulation of molecular evolution. “Substitution model” indicates the implemented models of non-coding DNA evolution [“All” means that all the reversible nucleotide substitution models are considered, JC, …, GTR], codon models [“ECMs” indicates empirical codon models, MG94 model refers to Muse and Gaut (1994) and HB model refers to Halpern and Bruno (1998)] and amino acid models [“+F” indicates that amino acid frequencies can be modeled]. “Rate variation” includes proportion of invariable sites “+I” and substitution rate heterogeneity across sites according to a gamma distribution “+G.” “Genome” indicates the consideration of genome evolution through region-specific substitution models*.

a *Coding sequences are simulated by nucleotide substitution models, avoiding stop codons*.

b *dN/dS can vary across codons*.

c *dN/dS can vary across branches*.

In order to improve the fitting to real data, current trends in the development of DNA substitution models involve non-reversible (asymmetric) and non-stationary (nucleotide composition can change over time) matrices (e.g., Boussau and Gouy, 2006; Jayaswal et al., 2011), or even consider neighbor interactions (Lunter and Hein, 2004), that can lead to more accurate phylogenetic inferences (Boussau and Gouy, 2006; Kaehler et al., 2015). However, these models have not been implemented yet in the most popular phylogenetic software packages due to their implicit complexity. In addition to the proposal of novel substitution models, future research should also address the implementation of these complex models in methods and programs for substitution model choice and phylogenetic inference.

## Trends in codon substitution models

Sites within codons evolve at different rates and, consequently, they should not be equally treated (Shapiro et al., 2006). Indeed, considering molecular evolution at the codon level allows us to incorporate more realistic evolutionary patterns for each codon position. Actually, evolutionary inferences based on the modeling of codon evolution are more robust than those derived from empirical amino acid models (Benner et al., 1994; Seo and Kishino, 2008).

Interestingly, mutations at the codon level can be classified as synonymous (silent) and non-synonymous (amino-acid replacing), which provides a measure of selective pressure at the molecular level (molecular adaptation). Then, the first codon substitution models included the non-synonymous and synonymous substitution rates, *dN* and *dS* respectively (Muse and Gaut, 1994), or the *dN/dS* ratio (Goldman and Yang, 1994). Despite the large number of (frequently ignored) considerations that should be made for the analysis and interpretation of *dN/dS* [i.e., *dN/dS* at the codon level can be biased if recombination is ignored (Anisimova et al., 2003; Arenas and Posada, 2010, 2014a) or if nucleotide frequencies vary across sites (Arenas and Posada, 2014b) and *dN/dS* should be estimated from samples of different populations (Kryazhimskiy and Plotkin, 2008; Pellissier, 2015)], *dN/dS* is commonly estimated in evolutionary biology for testing hypothesis related to selective pressure (e.g., Yang and Bielawski, 2000; Perez-Losada et al., 2009, 2011; Lopes et al., 2014; Arenas, 2015b; Arenas et al., 2015b; Lopez-Bueno et al., 2015). *dN/dS* >1 indicates that substitutions in the protein-coding gene were enriched for those that altered the amino acids states, suggesting diversifying (positive) selection. By contrast, *dN/dS* < 1 and *dN/dS* = 1 can be interpreted as purifying (negative) selection and neutral evolution, respectively.

Additional codon models have been proposed in order to better fit particular codon datasets. The GY94 codon model (Goldman and Yang, 1994) was extended to consider different nucleotide models (e.g., GTR; Kosakovsky Pond and Frost, 2005; Pond and Frost, 2005; Pond and Muse, 2005; Arenas and Posada, 2014b), *dN/dS* variation across sites (Yang et al., 2000; Anisimova et al., 2001; Kosakovsky Pond and Frost, 2005; Yang, 2007) and across branches (e.g., Pond and Frost, 2005; Yang, 2007; Dutheil et al., 2012). Other codon models implement information about the physicochemical properties of the encoded amino acids (Wong et al., 2006). Additionally, several empirical codon models—based on large databases of coding data—have been proposed (Schneider et al., 2005; Doron-Faigenboim and Pupko, 2007; Kosiol et al., 2007) and also codon models that consider codon bias (McVean and Charlesworth, 1999; Nielsen et al., 2007; Yang and Nielsen, 2008) or the effects of different GC contents (Misawa, 2011). Here, a promising trend is the emergence of mutation-selection models (e.g., Halpern and Bruno, 1998; Yang and Nielsen, 2008; Rodrigue et al., 2010) that attempt to integrate complex selection patterns into the mutation process and that outperform simple neutral models (Lawrie et al., 2011). Note that the interplay of mutational biases and weak selection can be complex, where constrained sites can evolve faster than neutral sites (McVean and Charlesworth, 1999; Lawrie et al., 2011). The consideration of these effects in substitution models of evolution can improve the identification of functional regions and the fitting to the data (Lawrie et al., 2011). For technical aspects on codon models the reader is referred to Anisimova and Kosiol (2009) and Cannarozzi and Schneider (2012).

Therefore, codon models allow us to perform accurate evolutionary analysis and to explore signatures of molecular adaptation. However, a problematic technical aspect regarding the implementation of these models is their large exchangeability matrices (61 × 61, note that stop codons are excluded). As a consequence, large amounts of data are needed to generate statistically well-supported empirical codon matrices and the computational burden is heavy. Fortunately, research on codon-based algorithm optimization is already generating new evolutionary tools to simulate (e.g., Fletcher and Yang, 2009; Arenas, 2012; Arenas and Posada, 2012) and analyze (e.g., Pond et al., 2005; Gil et al., 2013; Arenas et al., 2015a; Zoller et al., 2015) codon evolution (see Table 1), although further work is still required in this regard (e.g., the implementation of parallel computing of probability matrices).

The future of codon models will probably be related to the development of more complex models to better fit real data. In this concern, in addition to the development of new empirical models, codon models may follow two interesting trends. First, the consideration of heterogeneity along the sequence and over time, where different sites/regions and time periods could evolve under different models (Arenas, 2015a; Zoller et al., 2015). Note that these partition schemes can be very realistic, for example by considering different models for coding and non-coding regions. Moreover, it is known that codon models based on different codon frequencies across sites can bias *dN/dS* estimates (Arenas and Posada, 2014b), and Zoller et al. (2015) recently found that a two-partition codon model resolves a phylogeny better than a one-partition codon model. Consequently, methods and software to identify the best codon substitution model for particular codon regions (i.e., following Bao et al., 2008; Delport et al., 2010) and time periods are demanded. A second trend may be the consideration of structural information of proteins in codon models. Information derived from the protein function and from the protein folding stability [i.e., considering energy functions (Grahnen et al., 2011; Liberles et al., 2012; Arenas et al., 2015c)] of the encoded proteins could be considered in codon models. However, these implementations would require large computational costs if the protein structure varies with time or if more than one protein structure is needed to represent the encoded proteins of the dataset.

## Trends in amino acid substitution models

Substitution models of amino acid evolution intend to mimic the evolution of protein data, which is crucial for testing a variety of hypotheses such as selection toward novel proteins (e.g., Fares et al., 2002), rate of protein evolution (e.g., Alvarez-Ponce and Fares, 2012), role of protein function on protein evolution (e.g., Liberles et al., 2011), evolutionary aspects of protein-protein interaction networks (e.g., Alvarez-Ponce and Fares, 2012), multiple sequence alignment based on protein evolution (Zhao and Sacan, 2015), or phylogenetic tree and ancestral protein reconstructions (e.g., Perez-Jimenez et al., 2011). Substitution models of amino acid evolution can be classified in two major groups: (i) empirical models—based on large protein databases—[e.g., CpRev (Adachi et al., 2000), Dayhoff (Dayhoff et al., 1978), DayhoffDCMUT (Kosiol and Goldman, 2005), HIVb (Nickle et al., 2007), HIVw (Nickle et al., 2007), JTT (Jones et al., 1992), JonesDCMUT (Kosiol and Goldman, 2005), LG (Le and Gascuel, 2008), Mtart (Abascal et al., 2007), Mtmam (Yang et al., 1998), Mtrev24 (Adachi and Hasegawa, 1996), RtRev (Dimmic et al., 2002), VT (Müller and Vingron, 2000), WAG (Whelan and Goldman, 2001), see Table 1 for their implementation in phylogenetic software], and (ii) parametric models—based on parameters that describe protein evolution—(Rastogi et al., 2006; Liberles et al., 2012).

An empirical amino acid substitution model consists of a 20 × 20 matrix of exchangeability rates and 20 amino acid frequencies. This simplicity (compared to other amino acid models such as those based on structural constraints) leads to advantages but also pitfalls. Empirical models can be incorporated into the commonly used likelihood functions implemented in the standard phylogenetic software by assuming site-independence (all sites evolve under the same model). Heterogeneous evolution can also be modeled by specifying different empirical models for different partitions (i.e., sites or domains) of the protein sequence (Halpern and Bruno, 1998; Lartillot and Philippe, 2004; Zoller and Schneider, 2013). However, a given real dataset may not be properly represented by any of the currently available empirical models. For example, Keane et al. (2006) found that the best fitting empirical model for two large proteobacteria and archaea protein datasets was originally derived from retroviral *Pol* proteins.

In order to provide an alternative to empirical models, constraints on the protein folding have been considered to generate parametric amino acid substitution models that have led to significant improvements (with respect to empirical models) when fitting real data (e.g., Taverna and Goldstein, 2000; Parisi and Echave, 2005; Goldstein, 2011; Grahnen et al., 2011; Wilke, 2012; Arenas et al., 2013, 2015c; Bordner and Mittelmann, 2013). However, these models are not well-established yet in the evolutionary analysis of protein data because the commonly used evolutionary frameworks implement likelihood functions that cannot deal with site-dependence. Consequently, a current trend in structurally constrained substitution models is to generate site-specific matrices that can be incorporated into common phylogenetic frameworks (Arenas et al., 2015c).

New empirical models are emerging to represent protein families (e.g., Cox and Foster, 2013); similarly, new parametric models are appearing to account for different evolutionary processes across sites and over time—covarion models—(Usmanova et al., 2015), epistatic fitness landscapes (Usmanova et al., 2015), or complex structural constraints (Bordner and Mittelmann, 2013; Arenas et al., 2015c). Nevertheless, the large variety and complexity of current amino acid substitution models cause problems when implemented. As noted, complex amino acid models (such as those based on protein folding stability) are not established yet in popular phylogenetic software due to their implicit complexity. In addition, the user often restricts the candidate substitution models to those empirical models implemented in common substitution model choice programs (e.g., Abascal et al., 2005), which may lead to severe incongruences (Keane et al., 2006).

Therefore, although current research on amino acid substitution models is providing more sophisticated models, these models are usually not applied by evolutionary biologists because they are not implemented in evolutionary frameworks and, consequently, these models are often forgotten. In order to consider complex substitution models in model selection and in evolutionary analysis, an alternative strategy can be the approximate Bayesian computation (ABC) approach (Beaumont, 2010; Csilléry et al., 2010; Sunnaker et al., 2013; Lopes et al., 2014; Arenas, 2015a). Basically, simulated data under different complex models are contrasted with real data through multiple regression adjustments to identify the model that best fits the real data, and to estimate the parameter values of the model corresponding to the studied dataset (see Lopes et al., 2014 for an example of ABC using complex codon models).

## Conclusions

The modeling of genome evolution is nowadays important in population genomics and phylogenomics (Kumar et al., 2012; Librado et al., 2014). In that regard, as noted above, a variety of substitution models are known to mimic the evolution of coding and non-coding data. Importantly, the evolutionary process may differ between genomic regions because molecular evolution is often highly heterogeneous (Shapiro et al., 2006; Bofkin and Goldman, 2007; Arbiza et al., 2011) and, consequently, genome evolution should be mimicked with specific substitution models for each genomic region (Arbiza et al., 2011; Dalquen et al., 2012; Arenas and Posada, 2014b), see software implementation of partition models in Table 1. However, there is a need for methods to identify the regions that can better fit with a single substitution model. A strategy to perform this task could emulate genetic algorithms for the detection of recombination breakpoints based on phylogenetic tree incongruence (Fitch and Goodman, 1991; Grassly and Holmes, 1997; Kosakovsky Pond et al., 2006) assuming that different substitution models can also lead to significant signatures of phylogenetic tree incongruence (Minin et al., 2003; Lemmon and Moriarty, 2004). Then, these partitioning schemes could be evaluated with tools such as *PartitionFinder* (Lanfear et al., 2012). Additionally, a realistic modeling may involve not only region-specific models, but also branch-specific models (Ho, 2009). However, in practice only large datasets could provide sufficient statistical support for identifying the best models at those levels.

It seems clear that future research on substitution models of evolution will involve the development of more sophisticated and realistic substitution models. For example, the increasing amount of genomic data will probably require, in addition to partitioning, the development of complex mixture models where each site/region can be modeled with more than one substitution model. Concomitantly, efforts are needed to design and implement more robust methods to evaluate, compare and apply these complex substitution models.

## Funding

This work was supported by the Portuguese Government through the FCT Starting Grant IF/00955/2014.

### Conflict of interest statement

The author declares that the research was conducted in the absence of any commercial or financial relationships that could be construed as a potential conflict of interest.
